# Genotyping the hepatitis B virus with a fragment of the HBV DNA polymerase gene in Shenyang, China

**DOI:** 10.1186/1743-422X-8-315

**Published:** 2011-06-22

**Authors:** Ying Ma, Yang Ding, Feng Juan, Xiao Guang Dou

**Affiliations:** 1 Department of Neurology, Shengjing Hospital of China Medical University, Shenyang 110817, China; 2 Department of Infectious Disease, Shengjing Hospital of China Medical University, Shenyang 110817, China

**Keywords:** Hepatitis B virus, polymerase gene, mutation, genotype

## Abstract

The hepatitis B virus (HBV) has been classified into eight genotypes (A-H) based on intergenotypic divergence of at least 8% in the complete nucleotide sequence or more than 4% in the S gene. To facilitate the investigation of the relationship between the efficacy of drug treatment and the mutation with specific genotype of HBV, we have established a new genotyping strategy based on a fragment of the HBV DNA polymerase gene. Pairwise sequence and phylogenetic analyses were performed using CLUSTAL V (DNASTAR) on the eight (A-H) standard full-length nucleotide sequences of HBV DNA from GenBank (NCBI) and the corresponding semi-nested PCR products from the HBV DNA polymerase gene. The differences in the semi-nested PCR fragments of the polymerase genes among genotypes A through F were greater than 4%, which is consistent with the intergenotypic divergence of at least 4% in HBV DNA S gene sequences. Genotyping using the semi-nested PCR products of the DNA polymerase genes revealed that only genotypes B, C, and D were present in the 50 cases, from Shenyang, China, with a distribution of 11 cases (22%), 25 cases (50%), and 14 cases (28%) respectively. These results demonstrate that our new genotyping method utilizing a fragment of the HBV DNA polymerase gene is valid and can be employed as a general genotyping strategy in areas with prevalent HBV genotypes A through F. In Shenyang, China, genotypes C, B, and D were identified with this new genotyping method, and genotype C was demonstrated to be the dominant genotype.

## Background

Hepatitis B virus (HBV) infection is a substantial public health problem, with approximately 400 million virus carriers worldwide [1]. The infection can cause acute and chronic liver diseases, including cirrhosis and hepatocellular carcinoma (HCC) [1]. HBV has been classified into eight genotyped, designated as A-H, based on intergenotypic divergence of at least 8% in the complete nucleotide sequence or more than 4% in the S gene [2-8]. HBV genotypes have distinct geographical distributions and correlate with the severity of liver diseases. HBV genotype C is associated with more severe liver diseases than genotype B [9-11], and patients infected with genotype D appear to have a higher incidence of HCC [12], a higher risk for HBV recurrence, and a higher mortality rate after liver transplantation [13] than patients with genotype A. In addition, patients with HBV genotypes C and D have a lower response rate to treatment with IFN-α compared to those with genotypes A and B [9]. Genotype may also influence the emergence of lamivudine resistance mutations, which appear to be more strongly associated with genotype A than genotype D [14,15]. Therefore, HBV genotyping is of great importance in guiding treatment, improving vaccination, and controlling liver diseases.

In the past, genotyping was mostly performed on the full-length nucleotide sequence or the S gene sequence [2-8]. In order to facilitate the study of drug treatments, particularly how lamivudine resistance develops from polymerase gene mutations [14,15], we established a new genotyping method using a fragment of the HBV DNA polymerase gene that also slightly overlaps with the S gene. This genotyping method differs from past methods and aids in investigating the relationship between the efficacy of drug treatment and muations in specific genotypes of HBV.

## Methods

### Serum sample collection

Serum samples were collected with consent from patients and in accordance with Chinese State Ethics Regulation. This study included 33 patients with chronic hepatitis B with clinical symptoms (22 males, 11 females, mean age 31.79 years) that were to receive anti-virus treatment of lamivudine and 20 asymptomatic carriers (12 males, 8 females, mean age 29.5 years) that were enrolled as a control group. Chronic infection was defined as the detection of hepatitis B surface antigen (HBsAg) for at least 6 months. Among these samples, we found no co-infections with either human immunodeficiency virus or hepatitis C virus. All patients were born in Shenyang, China. The serum samples were collected and stored at -70°C.

### Primer design

The genome of HBV consists of four open reading frames, including the envelope gene (PreS/S), the core gene (PreC/C), the polymerase gene, and the gene encoding the transactivating protein X (X). Because of the compact organization of the genome, the complete PreS/S gene, part of the PreC/C gene and the X gene overlap with the polymerase gene. The S gene (nucleotides 155 to 833) encodes the major envelope proteins [16,17].

With the aid of DNASTAR software, we designed three primers to the sequence of the HBV DNA polymerase gene (accession number AF100309), named HBV381 (nt381-402), HBV840 (nt840-861), and HBV801 (nt801-822). The primers were synthesized (TaKaRa Biotechnology Co., Ltd., China) with the sequences of 5"-TGCGGCGTTTTATCATCTTCCT-3", 5"-GTTTAAATGTATACCCAAAGAC-3", and 5"-CAGCGGCATAAAGGGACTCAAG-3", respectively. Two pairs of semi-nested polymerase chain reaction (PCR) primers (HBV381/HBV840 and HBV381/HBV801) were utilized in the amplification reaction while primer HBV381 was used in the sequencing reaction. The amplified fragment of the polymerase gene overlaps slightly with the S gene.

### Polymerase gene fragment amplification and sequencing

HBV DNA was extracted from 55 serum samples obtained from infected patients. The fragment of the polymerase gene was amplified by semi-nested PCR with two rounds of amplification. The reaction volume was 50 μl. The first round of amplification was performed with an initial 5 min denaturing step at 94°C, followed by 30 cycles of denaturing for 45 s at 94°C, annealing for 30 s at 50°C, and elongation for 90 s at 72°C, with a final extension period of 10 min at 72°C using primers HBV381 and HBV840. The second round of amplification was performed with an initial 5 min denaturing step at 94°C, followed by 30 cycles of denaturing for 45 s at 94°C, annealing for 30 s at 55°C, and elongation for 60 s at 72°C, with a final extension period of 10 min at 72°C using primers HBV381 and HBV801. The reaction products of the semi-nested PCR were visualized on a 2% agarose gel stained with ethidium bromide. The semi-nested PCR reaction products were subjected to purification and sequencing using the HBV381 primer through a commercial company (Shanghai GeneCore Bio Technologies Co., Ltd. Shanghai, China) on an ABI sequencing system.

### Homology and phylogenetic analyses

The eight standard full-length nucleotide sequences of HBV DNA were obtained from GenBank (NCBI), including genotypes A (accession number AY128092), B (AB073858), C (AF461359), D (AY090453), E (X75664), F (X75663), G (AF405706), and H (AY090460). Nucleotide sequences of approximately 262 base pairs (bp) from the primer HBV381 were used for homology and phylogenic analysis. The eight standard nucleotide sequences of the HBV DNA genotypes, the predicted sequence of the semi-nested PCR products, and the actual sequences of the products were analyzed in pairs using CLUSTAL V (DNASTAR).

### Statistical analysis

The genotyping data were analyzed using a χ^2 ^test (SAS software, Cary, NC).

## Results

### Most HBV patients screen positive for the HBV polymerase gene by semi-nested PCR

**Figure 1:**
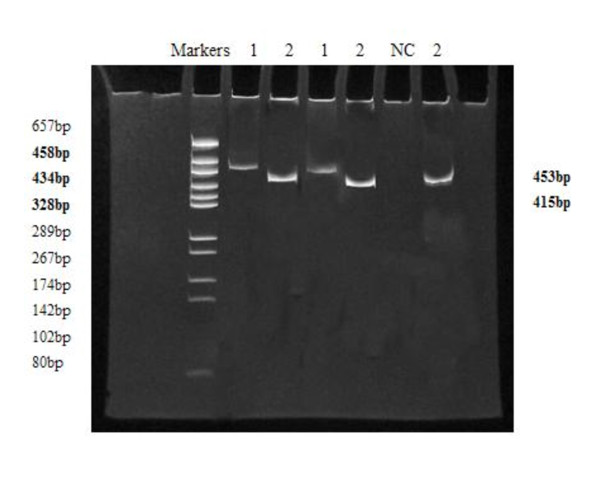
**Polyacrylamide gel electrophoresis of the reaction products of semi-nested PCR and DNA Markers**. The first round of amplification products (1), the second round of amplification products (2), and the negative control (NC) are shown.

The purpose of the semi-nested PCR was to screen for polymerase gene-positive patients. The PCR reaction products were the expected size (415 bp, Figure 1). In total, 50 samples (91%) were positive for polymerase gene fragments, 17 (85%) of which were from asymptomatic carriers and 33 (100%) of which were from patients with chronic hepatitis B. All the positive semi-nested PCR reaction products were then sequenced.

### Differences between genotypes (A-F) by analyzing the homology and phylogeny of the fragments intercepted from eight standard nucleotide sequences

**Figure 2:**
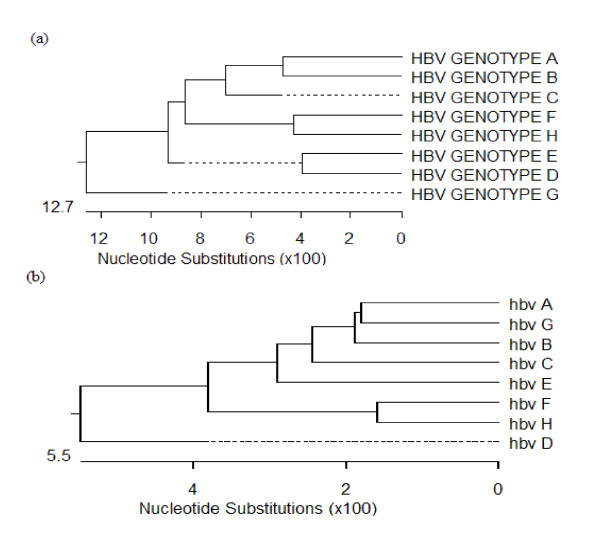
**(a) Phylogenetic tree of the full-length nucleotide sequences of the eight HBV DNA standard genotypes (HBV GENOTYPE A-H)**. (b) Phylogenetic tree of the nucleotide sequences of the fragments of the eight HBV DNA standard genotype polymerase genes (hbv A-H).

Homology and phylogenetic analysis of the eight standard genotype full-length nucleotide sequences were performed using CLUSTAL V. The difference in nucleotide sequence between genotypes D and E was 7.9%, which was the smallest among all the genotypic differences. The largest difference in nucleotide sequence was found between genotypes B and H and between genotypes G and H, both of which were 15.9% (Figure 2a, Table 1). The results were consistent with an intergenotypic divergence of at least 8% in the complete nucleotide sequence [2-7]. Homology and phylogenetic analysis of the semi-nested PCR fragments (262 bp) from the DNA polymerase genes of the standard strains revealed that the differences between genotypes A and G, B and G, and F and H were 3.5%, 3.1%, and 3.1%, respectively (Figure 2b, Table 2). However, all the other differences between pairs of genotypes (A-F), such as between genotypes A and B, A and C, B and C, etc., were greater than 4%, consistent with an intergenotypic divergence of at least 4% in HBV DNA S gene sequences [8].

**Table 1:** Homology of eight HBV DNA standard genotype full-length nucleotide sequences (%)

Genotype	HBV A	HBV B	HBV C	HBV E	HBV F	HBV G	HBV H	HBV D
HBV A	***	90.3	91.2	88.9	85.7	87.4	84.9	89.2
HBV B	**9.5**	***	89.3	87.1	84.8	85.5	84.3	87.6
HBV C	**8.5**	**10.3**	***	88.5	85.5	86.2	85.4	88.0
HBV E	**10.5**	**12.3**	**10.5**	***	85.1	88.5	84.6	91.5
HBV F	**14.3**	**15.5**	**14.4**	**14.7**	***	84.3	91.3	85.2
HBV G	**11.8**	**13.9**	**13.1**	**11.3**	**15.5**	***	84.0	87.6
HBV H	**15.1**	**15.9**	**14.5**	**15.2**	**8.6**	**15.9**	***	84.8
HBV D	**10.5**	**12.1**	**10.4**	**7.9**	**14.8**	**12.1**	**15.5**	***

Bold numbers below *** indicate intergenotypic divergence of the two HBV DNA standard genotypes.

**Table 2:** Homology of the fragments of the eight HBV DNA standard genotype polymerase gene nucleotide sequences (%)

Genotype	HBV A	HBV B	HBV C	HBV D	HBV E	HBV F	HBV G	HBV H
HBV A	***	95.4	93.5	91.2	92.0	89.3	96.6	91.6
HBV B	**4.4**	***	95.8	92.3	94.6	92.3	96.6	93.9
HBV C	**6.0**	**4.0**	***	94.3	94.6	90.8	93.9	92.0
HBV D	**8.1**	**7.2**	**5.6**	***	92.7	91.6	92.0	92.0
HBV E	**8.1**	**5.6**	**5.2**	**6.4**	***	92.7	93.5	92.3
HBV F	**10.3**	**7.2**	**8.5**	**8.1**	**7.2**	***	91.2	96.9
HBV G	**3.5**	**3.1**	**5.6**	**7.2**	**6.4**	**8.1**	***	93.5
HBV H	**8.5**	**6.4**	**8.1**	**7.7**	**7.2**	**3.1**	**6.4**	***

Bold numbers below *** indicate intergenotypic divergence of the two fragments of the P gene nucleotide sequences.

### Genotypes B, C and D by genotyping the sequencing results of the semi-nested PCR products with the intercepted fragments of the standard genotypes polymerase gene

**Figure 3:**
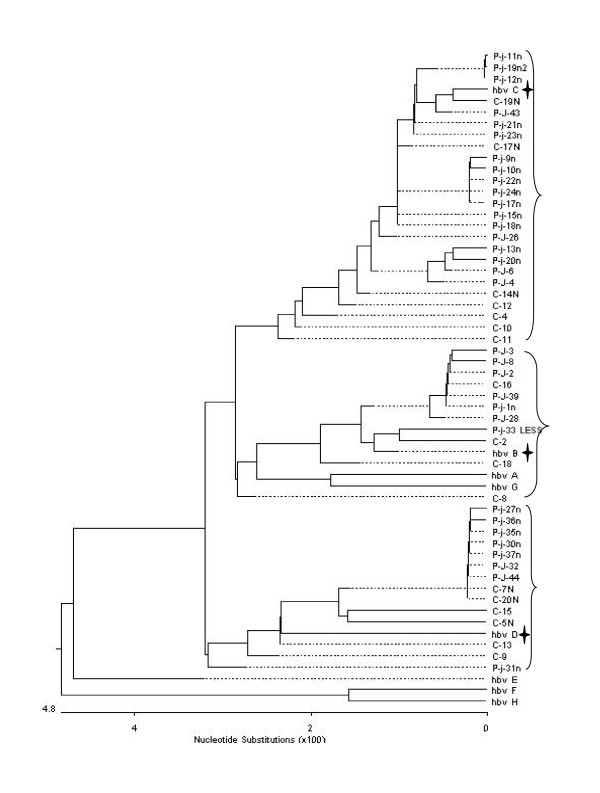
**Phylogenetic tree of the nucleotide sequences of the fragments of the semi-nested PCR products and HBV DNA standard genotype polymerase genes**. Patients with chronic hepatitis B (P-), asymptomatic carriers of HBV (C-), and the fragments of the standard genotype polymerase genes (hbv A-H) are shown. Only genotypes B, C, and D were detected in the 50 HBV DNA-positive cases.

Only genotypes B, C, and D were detected in the 50 HBV DNA-positive cases, occurring in 11 (22%), 25 (50%), and 14 cases (28%), respectively (Figure 3, Table 3). The proportions of these genotypes were significantly different ((p = 0.000) and the proportions of genotypes C and B and genotypes C and D were significantly different (p < 0.05). Of the 33 patients with chronic hepatitis B, genotypes B, C, and D were detected in 7 (21%), 18 (55%), and 8 cases (24%), respectively. The proportions of these genotypes were significantly different (p = 0.001) and the proportions of genotypes C and B and genotypes C and D were significantly different (p < 0.05). Of the 17 asymptomatic carriers of HBV, genotypes B, C, and D were detected in 4 (24%), 7 (41%), and 6 cases (35%), respectively. The proportions of these genotypes were not significantly different (p = 0.053). There was no significant different in the proportions of these genotypes between patients with chronic hepatitis B and HBV asymptomatic carriers. While the proportion of genotype D was similar to genotype B and slightly lower than genotype C in all the groups analyzed, the proportion of genotype D was 35% in asymptomatic carriers of HBV and only 24% in patients with chronic hepatitis B (p = 0.410). Genotypes A, E, F, G, and H were not detected in any cases.

**Table 3:** Genotypes of all 50 HBV DNA-positive cases

Genotype	Total patients	CHB	ACHB	***P***^***a***^**value**
Genotype B	11 (22%)	7 (21%)	4 (24%)	0.629
Genotype C	25 (50%)	18 (55%)	7 (41%)	0.370
Genotype D	14 (28%)	8 (24%)	6 (35%)	0.410
*P *value	0.000 BC 0.004 CD 0.024	0.001 BC 0.005 CD 0.012	0.053 BC 0.271 BD 0.452	

ACHB: asymptomatic carriers of HBV; CHB: chronic hepatitis B; BC: *P *value between genotypes B and C; BD: *P *value between genotypes B and D; CD: *P *value between genotypes C and D; *P*^*a*^: *P *value between patients with chronic hepatitis B and HBV asymptomatic carriers.

## Discussion

Simple and effective alternatives to the gold standard method for genotyping HBV of sequencing the entire HBV genome have been developed, including restriction fragment length polymorphism [18], multiplex PCR with type-specific primers [19-21], and others. However, most of these newer methods are based on analyzing the S gene. Here we have developed a novel genotyping method based on a segment of the HBV DNA polymerase gene. Using CLUSTAL V, we demonstrated that the eight standard genotypes selected from the GenBank (NCBI) had intergenotypic divergence of at least 8% in their complete nucleotide sequences [2-7]. We then confirmed that the semi-nested PCR products from the DNA polymerase gene had intergenotypic divergence of at least 4%, which is in accordance with the HBV DNA S gene sequence [8], except between genotypes A and G, B and G, and F and H. However, the intergenotypic divergence among genotypes A through F were higher than 4% in our selected region. Genotypes F and H have only been detected in Central and South America, and genotype G has been identified in France, Germany, Mexico, and the United States. Genotype A is more prevalent in northwestern Europe, North America, India, and sub-Saharan Africa. Only genotypes B, C, D, and A have been found in China [22-30]. Therefore, the fragment of HBV DNA polymerase gene can be used for genotyping hepatitis B in China. This genotyping method can also be used to predict antiviral therapeutic response among HBV genotypes and the development of drug resistant due to mutations. It is a valuable tool for guiding the treatment of lamivudine-resistant HBV in the clinical setting [27-30].

We analyzed the nucleotide sequences of the semi-nested PCR products of the HBV DNA polymerase gene in the 50 patient samples using CLUSTAL V. Genotypes C, B and D were detected while genotypes A, E, F, G, and H were not. Half of the HBV DNA polymerase-positive samples were genotype C, making it the dominant genotype. It was also the major genotype (55%) in the 33 patients with chronic hepatitis B. The differences in the proportions of genotypes B, C, and D were not significant in the 17 asymptomatic carriers of HBV. The proportion of genotype D was similar to genotype B, and its proportion in asymptomatic carriers of HBV was slightly, but not significantly (p = 0.410), higher than that in patients with chronic hepatitis B. Our results suggest that genotype C is the dominant genotype among asymptomatic carriers and that genotype D may be more frequent in asymptomatic carriers than in patients with chronic hepatitis B.

## Conclusions

A new method of genotyping HBV via sequencing a fragment of its DNA polymerase gene is a valid strategy for genotyping hepatitis B in areas with a high prevalence of genotypes A through F. It provides a novel alternative to complete sequencing of the HBV genome and allows the study of the relationship between genotype and mutations of HBV DNA polymerase gene induced by antiviral therapy. Using this method, genotypes C, B, and D were identified in patients from Shenyang, China, and genotype C was demonstrated to be the dominant genotype. However, analysis of the difference in the proportions of the genotypes between asymptomatic carriers of HBV and patients with chronic hepatitis B, as well as the impact of genotype on therapeutic response and virus mutation, require further study.

## List of abbreviations

HBV: hepatitis B virus; PCR: polymerase chain reaction; HCC: hepatocellular carcinoma.

## Competing interests

The authors declare that they have no competing interests.

## Authors' contributions

YM and XGD designed, executed and coordinated the study. YM, YD and XGD contributed in the sample acquirement and laboratory analysis. YM, JF and XGD participated in the drafting of the manuscript and literature search. All authors read and approved the final manuscript.
